# The Role of ERK1/2 in the Development of Diabetic Cardiomyopathy

**DOI:** 10.3390/ijms17122001

**Published:** 2016-12-08

**Authors:** Zheng Xu, Jian Sun, Qian Tong, Qian Lin, Lingbo Qian, Yongsoo Park, Yang Zheng

**Affiliations:** 1 Cardiovascular Center, The First Hospital of Jilin University, Changchun 130021, China; wsxuzheng@163.com (Z.X.); sunjianemail@126.com (J.S.); tongqian187@aliyun.com (Q.T.); 2 Department of Pediatrics, Kosair Children’s Hospital Research Institute, University of Louisville, Louisville, KY 40202, USA; bioqian@163.com; 3 Department of Pharmacology & Toxicology, University of Louisville, Louisville, KY 40202, USA; q0lin002@louisville.edu; 4 Department of Basic Medical Sciences, Hangzhou Medical College, Hangzhou 310053, China; 5 College of Medicine & Engineering, Hanyang University, Seoul 04963, Korea

**Keywords:** diabetic cardiomyopathy, ERK1/2 MAPK, cardiac dysfunction, cardiac remodeling, histone deacetylase (HDAC), microRNAs

## Abstract

Diabetes mellitus is a chronic metabolic condition that affects carbohydrate, lipid and protein metabolism and may impair numerous organs and functions of the organism. Cardiac dysfunction afflicts many patients who experience the oxidative stress of the heart. Diabetic cardiomyopathy (DCM) is one of the major complications that accounts for more than half of diabetes-related morbidity and mortality cases. Chronic hyperglycemia and hyperlipidemia from diabetes mellitus cause cardiac oxidative stress, endothelial dysfunction, impaired cellular calcium handling, mitochondrial dysfunction, metabolic disturbances, and remodeling of the extracellular matrix, which ultimately lead to DCM. Although many studies have explored the mechanisms leading to DCM, the pathophysiology of DCM has not yet been fully clarified. In fact, as a potential mechanism, the associations between DCM development and mitogen-activated protein kinase (MAPK) activation have been the subjects of tremendous interest. Nonetheless, much remains to be investigated, such as tissue- and cell-specific processes of selection of MAPK activation between pro-apoptotic vs. pro-survival fate, as well as their relation with the pathogenesis of diabetes and associated complications. In general, it turns out that MAPK signaling pathways, such as extracellular signal-regulated kinase 1/2 (ERK1/2), c-Jun N-terminal protein kinase (JNK) and p38 MAP kinase, are demonstrated to be actively involved in myocardial dysfunction, hypertrophy, fibrosis and heart failure. As one of MAPK family members, the activation of ERK1/2 has also been known to be involved in cardiac hypertrophy and dysfunction. However, many recent studies have demonstrated that ERK1/2 signaling activation also plays a crucial role in FGF21 signaling and exerts a protective environment of glucose and lipid metabolism, therefore preventing abnormal healing and cardiac dysfunction. The duration, extent, and subcellular compartment of ERK1/2 activation are vital to differential biological effects of ERK1/2. Moreover, many intracellular events, including mitochondrial signaling and protein kinases, manipulate signaling upstream and downstream of MAPK, to influence myocardial survival or death. In this review, we will summarize the roles of ERK1/2 pathways in DCM development by the evidence from current studies and will present novel opinions on “differential influence of ERK1/2 action in cardiac dysfunction, and protection against myocardial ischemia-reperfusion injury”.

## 1. Introduction

Diabetes mellitus (DM) is one of the world’s most common metabolic disorders. The number of global diabetic patients rose to 415 million in 2015 [1], about 8.3% of the adult population [2]. From 2012 to 2015, DM and its complications were estimated to bring about 1.5 to 5.0 million deaths each year [1,3]. Cardiac dysfunction, namely diabetic cardiomyopathy (DCM), is one of the major complications that accounts for more than half of diabetes-related morbidity and mortality. Hyperglycemia and hyperlipidemia in diabetes mellitus cause cardiac oxidative stress, endothelial dysfunction, impaired cellular calcium handling, mitochondrial dysfunction, metabolic disturbances, and remodeling of the extracellular matrix, which ultimately lead to DCM [4,5,6]. The manifestation of DCM includes and begins with diastolic dysfunction, reduced left ventricular function, early heart failure associated with abnormal myocardial energy metabolism, and may encompass late myocardial hypertrophy, fibrosis, and death [7]. The impaired diastolic function is the earliest abnormality in DCM, and systolic dysfunction supervenes only at later stages of DCM [8]. Although numerous studies have explored the potential mechanisms in DCM, the pathophysiology of DCM has not yet been fully clarified because of the multi-factorial pathophysiologic involvement in the development of DCM. In fact, many researchers have realized the association of mitogen-activated protein kinase (MAPK) involvement during DCM development. MAPK signaling pathways, such as extracellular signal-regulated kinase 1/2 (ERK1/2), c-Jun N-terminal protein kinase (JNK) and p38 MAP kinase, are demonstrated to be actively involved in myocardial dysfunction [9,10], hypertrophy [11,12,13], fibrosis [14,15,16] and heart failure [17,18,19]. As one MAPK family member, ERK1/2 has been known to be involved in cardiac hypertrophy [20,21]. Recently, many studies have demonstrated that the ERK1/2 signaling pathway plays a crucial role in the development of accelerated DCM [22,23,24]. In this review, we will summarize the emerging roles of the ERK1/2 pathway in DCM development, on the basis of the evidence from recent studies, and will present a novel opinion on the role of ERK1/2 in the heart in its disease state.

## 2. Mitogen Activated Protein Kinase (MAPK) Pathway

MAPK enzymes are highly conserved among eukaryotes, and the genealogy of human MAPKs can be traced back to evolutionary divergences in primitive eukaryotes. There are four classical subfamilies of the MAPK in humans: ERK1/2 (MAPK 3/1), p38 (MAPKs 11–14), JNK (MAPKs 8–10), and ERK5 (big MAPK 1 or MAPK 7). The MAPK family is activated via a sequential phosphorylation cascade, recruiting MAPK kinase (MEK) and MAPK [25]. Human MAPKs respond to an array of different stimuli. For example, ERK1/2 and ERK5 can be triggered by several agonists and cytokines [26,27,28]. ERK1/2 is also well known to be activated by growth factors. JNK and p38 can also be initiated by different growth factors and G-protein coupled receptors (GPCR) [27,29]. Under these stresses, highly conserved three-tiered serial phosphorylation cascades begin when MEK kinases phosphorylate and activate second tier MEKs. The latter dually phosphorylates the activation loop of their corresponding MAPKs on the characteristic threonine-X-tyrosine motif, where X is a variable amino acid residue (glutamic acid in ERK1/2, glycine in p38, and proline in JNK) to influence dynamic myocardial growth or adaptation [30,31]. Crosstalk between each member of MAPKs is considerable. There could be both stimulatory and inhibitory interactions within and across different MAPKs. Reactive oxygen species (ROS) have been reported to activate ERKs, JNKs, and p38 MAPKs, but the mechanism by which ROS can activate these kinases remains to be determined.

MAPKs participate in cardiac physiology and various cardiovascular diseases, which initiates accruing studies for exploring the pharmacological/genetic blockers/activators and downstream targets of the MAPK pathway in the heart [32,33]. The MAPKs can phosphorylate a great variety of protein kinases and transcription factors to regulate cellular development, survival, and death [10,30,34,35,36]. The activation of MAPK signaling by stress is normally ceased by MAPK phosphatases (MKPs) and tyrosine phosphatases [37,38]. Such cellular balance of MAPK signaling helps to immediate strengthening of cardiac contractility without hypertrophy [37]. In addition, mitochondrial signaling and other protein kinases have also been reported to regulate MAPK at upstream and downstream to modulate cardiac physiology and pathophysiology [31].

## 3. ERK1/2 Signaling Pathway

**Figure 1:**
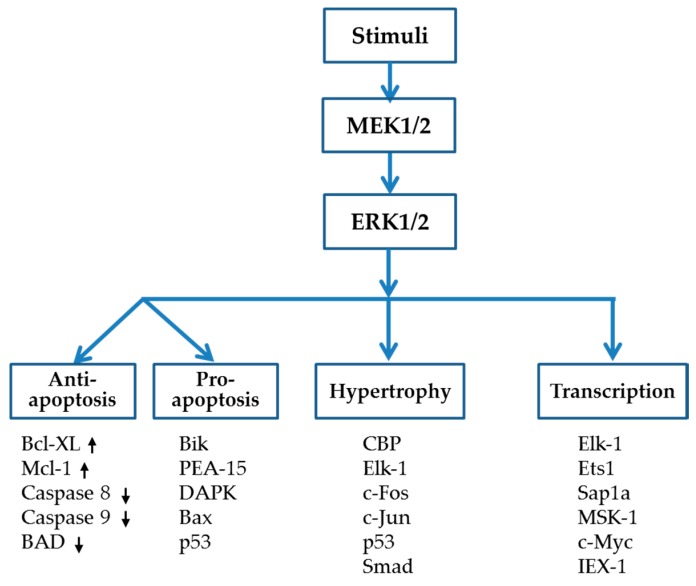
Intracellular targets of ERK1/2.

The ERK1/2 activation cascade, also known as the Ras-Raf-MEK-ERK pathway, has been extensively investigated. Activation of the heterotrimeric G protein Ras leads to activation of the MEK kinase Raf, which in turn phosphorylates and activates MEK, while the stimulated MEK then phosphorylates ERK1 and ERK2 at threonine 202 and tyrosine 204 to activate the enzymes [25]. ERK1 and ERK2 are 84% identical in sequence and have tended to be in use interchangeably [25,39]. The double bands on electrophoresis gels (phospho-ERK1 and phospho-ERK2) are usually taken together as representing total ERK activity triggered by numerous stimuli. ERK1/2 contains unique N- and C-terminal extensions that provide signaling specificity. In addition, the ERK1/2 family contains a 31-amino-acid-residue insertion within the kinase domain (kinase insert domain) that provides additional functional specificity [40]. Generally, activation of ERK1/2 participates in the regulation of meiosis, mitosis, and post-mitotic function [21], which are closely related to cell growth, proliferation, differentiation, migration, and survival. As shown in Figure 1, intracellular targets of ERK1/2, which affect transcription, apoptosis, and hypertrophy, include Elk-1, c-Fos, MSK-1, c-Myc, GATA4, Ets1/2, Mcl-1, Bcl-XL, IEX-1, and so on.

ERK1/2 signaling is classically triggered by activation of the small G-protein Ras at the myocyte menbrane, which then recruits and activates c-Raf, a MAP3K [41]. The activated MAP3K phosphorylates MEK 1/2, a MAP2K, to catalyze the phosphorylation of human ERK1/2 at Tyr204/187 and then Thr202/185, the motif of which locates within the activation loops [40,42]. Activation of ERK1/2 not only leads to the phosphorylation of numerous cytoplasmic targets, but also translocates ERK1/2 into the nucleus to activate target transcriptional factors as shown in Figure 1. A large amount of evidence reveals the role of the ERK1/2 signaling cascade in the development of DCM, most of which suggests that ERK1/2 activation plays a detrimental role in the process of oxidative stress, inflammation, remodeling and apoptosis in the diabetic heart. However, ERK1/2 has also been known to be a protective factor against myocardial infarction (MI) and ischemia/reperfusion (I/R) injury in diabetes due to its pro-survival effect [43,44,45]. These distinct effects of ERK1/2 might be related to the differences in the extent, subcellular compartmentalization and duration of ERK1/2 activity trigged by different stimuli [46]. It is reported that ERK1/2 signaling is mainly stopped by protein phosphatase 2A (PP2A) and/or dual-specificity phosphatases (DUSPs) induced dephosphorylation at tyrosine and/or threonine residues [21,47]. Activation of the ERK1/2 signaling cascade has long been implicated in mediating most stress stimuli-induced cardiac hypertrophy. As a result, many compounds have been developed that inhibit the ERK1/2 pathway and thus potentially attenuate cardiac growth and proliferation. The most widely used pharmacological agents are MEK-ERK inhibitors, PD98059 and U0126. Recently, more and more researchers pay attention to exploring the selective ERK1/2 inhibitor for fear of the potential side-effects of MEK-ERK inhibitors. In addition, the ERK1/2 double null mice model has been introduced to define the differential role of ERK1/2 in developing various heart diseases [48]. Instead of phosphorylating ERK1/2 at threonine 202 and tyrosine 204, autophosphorylation of ERK1/2 at a new site Thr188 has been specifically introduced to cause cardiac hypertrophy [49]. Finally, researchers have begun to study epigenetic regulation of the ERK1/2 signaling as a whole by histone deacetylases (HDACs) and/or HDAC inhibitors as well as microRNA (miRNA) to manipulate and therefore prevent different cardiac diseases.

## 4. ERK1/2, a Two-Edged Sword in DCM Development

In general, ERK1/2 is believed to be involved in the progression of DCM in vivo and in vitro. Despite the pro-apoptotic role of ERK1/2 in diabetes settings, the pro-survival effect of ERK1/2, which protects from cardiac injury induced by I/R and MI on the diabetic heart, has been noted.

### 4.1. Oxidative Stress

ROS play a major role in the initiation and development of DCM. Some studies made a putative hypothesis regarding the connection between ROS and ERK1/2 activation. The stimulation of endothelin 1 (ET-1) or phenylephrine (PE; α1-adrenergic receptor agonist) increased the ROS level significantly in cardiomyocytes. Meanwhile, ERK1/2 activity was also increased by the stimulation of ET-1 or PE. Therefore, antioxidant treatment of cardiomyocytes may suppress the growth in the level of ROS and block ERK1/2 activation, and then prevent the subsequent cardiac hypertrophy induced by these stimuli [50].

High glucose (HG) not only caused H9c2 cells to experience cytotoxicity, apoptosis, overproduction of ROS, and the decrease of mitochondrial membrane potential (MMP), but also upregulated the expression of p-ERK1/2. Some potential antioxidant agents, such as hispidin and sodium hydrogen sulfide markedly reduced p-ERK1/2. The ERK1/2 inhibitor (U0126) could also alleviate HG-induced cardiomyocyte injury, for example by increasing cell viability and by decreasing the number of apoptotic cells and ROS generation. These results suggest that ROS could be inhibited via inhibiting the ERK1/2 pathway [51].

### 4.2. Anti-Apoptosis vs. Pro-Apoptosis Influence of ERK1/2

Most of the studies implicate that ERK1/2 activation plays a detrimental pro-apoptotic role in the process of oxidative stress, inflammation, remodeling and apoptosis in the diabetic heart. Cardiomyopathy is a late consequence of initial diabetes-induced early cardiac responses. Cardiomyocyte apoptosis is one of the crucial components in early cardiac responses that may lead to devastating complications of cardiomyopathy. Many drugs were developed to prevent ERK1/2 activation in diabetic hearts and cardiomyocytes under diabetic conditions and ultimately to prevent DCM, following the observation that the inhibition of ERK1/2 may prevent cell death in HG-stimulated cardiomyocytes.

In Ni’s study, exploiting the *db*/*db* transgenic type 2 diabetes mellitus (T2DM) and streptozocin (STZ)-induced type 1 diabetes mellitus (T1DM) models, they tried to inhibit ERK1/2 and DCM development with mito-TEMPO which is a physicochemical compound, one of the superoxide dismutase (SOD) mimetics targeting mitochondrial ROS. Their results suggest that therapeutic inhibition of mitochondrial ROS by mito-TEMPO decreased adverse cardiac changes and mitigated myocardial dysfunction in diabetic mice. In particular, the study demonstrated that the protective effects of mito-TEMPO were associated with the down-regulation of ERK1/2 phosphorylation. In fact, administration of mito-TEMPO profoundly prevented ERK1/2 activation in diabetic hearts and cardiomyocytes under diabetic conditions. In addition, the inhibition of ERK1/2 prevented cell death in HG-stimulated cardiomyocytes [52].

In contrast, a considerable number of studies confirmed the anti-apoptosis effects of ERK1/2, which may represent a potential mechanism underlying cardiac protection in diabetes [53,54]. Besides the well-known effects of ERK1/2 related to cell apoptosis and hypertrophy, as the extracellular regulated protein kinases, ERK1/2 takes an active part in cell proliferation and differentiation. The up-regulation of ERK1/2 shows protection on I/R or MI in DCM. Activating ERK1/2 has been reported to participate in the cardioprotection of ischemic/pharmacological preconditioning/postconditioning against I/R injury [43,55,56,57]. However, diabetes inhibited the cardioprotection induced by preconditioning/postconditioning against I/R, which might be related to the reduced recovery of ERK1/2 [43,44,55]. In addition, Lambert et al. studied how Na_2_S therapy attenuates I/R injury in an ERK1/2-dependent manner. U0126 abolished the infarct sparing effects of Na_2_S therapy [45]. The protection of hydroxychloroquine in the heart during I/R injury was blocked in the presence of the ERK1/2 inhibitor, U0126 [58]. All of these studies showed the protective effects of ERK1/2 on I/R or MI in diabetes via enhancing the phosphorylation of the pro-survival kinase ERK1/2 [59].

Although ERK1/2 is a pro-survival factor in the MAPK family and contributes to the positive regulation of cell proliferation and differentiation under some conditions, ERK1/2 can function in a pro-apoptotic manner. The potential cause may be that ERK1/2 downstream substrates have distinct functions, and the targeted selection of ERK1/2 downstream substrates depends on cell type, nature of the stimuli, cellular compartments in which ERK1/2 is localized, and interaction between ERK1/2 and substrates [60]. The specificity of activation or inhibition of downstream effectors determines the consequence of ERK1/2 activation on cell survival, which is anti-apoptotic, but in some cases pro-apoptotic. Studies of the heart and the concrete mechanism by which ERK1/2 activation promotes apoptosis are lacking, and this subject needs further investigation [61].

In summary, ERK1/2 has a close relation to the pathological process of DCM. Contrary to the negative effects of active ERK1/2 in diabetes settings, the pro-survival effect of ERK1/2 protects against cardiac injury induced by I/R and MI in the diabetic heart.

### 4.3. Hypertrophy

Cardiac hypertrophy usually occurs in the late stage of diabetes, which eventually lead to cardiac remodeling, dysfunction, and even heart failure.

One of most commonly used studies exploring the role of ERK1/2 in DCM consists of setting up the HG-induced cardiomyocyte hypertrophy model. Ding et al. insisted that there is an HG-induced pathologic influence on neonatal rat cardiomyocytes and myocardium tissue from diabetic rats. The results showed that HG increased cell size and hypertrophy gene expressions, and was accompanied by elevating the expression of ERK1/2 but not of p38 MAPK or JNK activity. Furthermore, the cardiac hypertrophic responses of all cells were decreased after the treatment of ERK1/2 inhibitor PD98059. Consistent changes of hypertrophy and ERK1/2 expressions were also observed in STZ-induced myocardium of diabetic rats [62]. It is believed that ERK1/2 activation in response to hyperglycemia results in cardiac hypertrophy [63,64].

### 4.4. Fibrosis

Myocardial fibrosis is another essential hallmark of DCM contributing to cardiac dysfunctions. In a recent study, authors checked the anti-fibrosis effect of resveratrol, an antioxidant. They observed that transforming growth factor beta (TGF-β) and p-ERK1/2 were elevated in the condition of HG, while the HG-induced up-regulation of TGF-β was suppressed by U0126, suggesting the involvement of ERK1/2 in HG-induced TGF-β expression [65,66]. Additionally, HG increased ERK1/2 activity in the cardiac fibroblasts in vitro. ERK1/2 inhibitor PD98059 or U0126 suppressed HG-induced fibroblast proliferation and expressions of collagen. These findings indicate that blocking ERK1/2 signaling may be an important measure leading to cardiac protection in diabetes [67].

### 4.5. FGF21 Functions on ERK1/2 Signaling

Fibroblast growth factor 21 (FGF21) has been identified as a potential metabolic regulator with specific effects on glucose and lipid metabolism. Glucolipotoxicity and insulin resistance play an important role in development and progression of diabetes and its complications such as DCM. The heart might be an FGF21 target, yet the actions of FGF21 in the heart under normal and diabetic conditions are not fully understood. Although the precise underlying signal transduction mechanisms caused by individual injuries remain to be established, FGF21-mediated protection against DCM development [23,24], myocardial I/R injury [68,69] and isoproterenol (ISO)-induced cardiac hypertrophy [70] have been demonstrated. Interestingly, Zhang et al. found that FGF21 protected the heart from cardiac apoptosis, remodeling and dysfunction in diabetic mice through ERK1/2 activation. In their study, palmitate downregulated, but FGF21 upregulated phosphorylation levels of ERK1/2 and p38 MAPK. Indeed, FGF21 prevents palmitate-induced cardiac apoptosis via upregulating the ERK1/2-dependent p38 MAPK signaling. Inhibition of ERK1/2 by PD98059 completely abolished FGF21-mediated prevention of diabetes-induced cardiac apoptosis. When STZ induced mice were treated with FGF21 and PD98059 simultaneously, FGF21-mediated protection from cardiac remodeling and dysfunction was significantly blocked by PD98059 treatment [23]. To confirm the salutary role of ERK1/2 in FGF21 signaling, more studies should be carried out which focus on the interaction of FGF21 signaling and ERK1/2 activation with and without other MAPK and AMPK activation.

In summary, ERK1/2 is involved in the modulation of various diabetes-induced cardiac pathological changes. However, performing a salutary influence during FGF21 signaling and I/R injury, ERK1/2 exerts the cardiac effect, from compensatory hypertrophy to myocardial apoptosis and remodeling. Thus, the intricate role of ERK1/2 signaling cascades should be taken into account so carefully as to be a potential therapeutic target for DCM.

## 5. Inhibition of MEK/ERK to Study the Function of ERK

### 5.1. Exploiting Pharmacologic Agents to Study ERK1/2 Function

As shown above, ERK1/2 is associated with many pathological processes. Consequently, the inhibition of ERK1/2 might present ameliorative effects on various cardiac dysfunctions. Until now, several inhibitors of the ERK1/2 signaling pathway have been developed and made commercially available. The most frequently studied inhibitors of ERK1/2 pathway are U0126 and PD98056 [23,24,53,62,66]. These agents are unusual as small molecule inhibitors since they are expected to demonstrate steady-state and non-competitive inhibition.

However, both U0126 and PD98056 inhibit all MEKs, which are upstream of ERKs [71]. Earlier assumptions that ERK1/2 is the only known downstream target of MEK kept researchers from seeking additional benefit from an ERK1/2 specific inhibitor [72]. It was not until ERK1- and ERK2-selective inhibitors were developed that the resistance of nonspecific MEK inhibitors was suspected of leading to an incomplete blockage of ERK1/2 signaling. In particular, one study shows that both U0126 and PD98059 block MEK5, the kinase that activates ERK5 in addition to ERK1/2. Despite similarities between ERK1/2 and ERK5, recent studies have revealed new distinctive features of the ERK5 pathway [71]. Thus, theoretically, both U0126 and PD98059 have the off-target effect on ERK1/2 [73]. Moreover, the resistance of Raf and MEK inhibitors frequently may cause the recovery of ERK signaling [72], suggesting an important benefit of an ERK1/2 selective inhibitor that could help the potency and durability of ERK1/2 pathway inhibition.

As shown in Table 1, some ERK1- and/or ERK2-selective inhibitors have been discovered, such as FR180204, SCH772984, and GDC-0994. However, their applications are limited. Ohori et al. found that FR180204 inhibited ERK1 and ERK2 with an IC50 value of 0.51 μm (*K*i = 0.31 μm) and 0.33 μm (*K*i = 0.14 μm), respectively, and failed to inhibit other kinases (recombinant MEK1, MKK4, IKKα, PKCα, Src, Syc and PDGFα from human). Based on the results of a Lineweaver–Burk analysis, FR180204 is classified as an ATP-competitive inhibitor [74]. Taglieri et al. found that p21-activated kinase-1 (Pak-1) in mice facilitates ISO-induced cardiac hypertrophy in association with activation of ERK1/2. At the same time, the administration of the ERK1/2 selective inhibitor FR180204 reduced the ERK1/2 phosphorylation and LV myocardial hypertrophy in ISO-treated WT and Pak-1-knockout (KO) mice [75]. Several studies revealed superiority of other ERK1/2-selective inhibitors, such as SCH772984 [76,77,78] and VTX-11e [79]. These two highly selective, ATP-competitive, preclinical inhibitors have a tight relationship in antagonizing the growth of tumors. In another study, GDC-0994 [80] was used to confirm ERK1/2 involvement in the process of unsaturated fatty acids inducing transcriptional regulation of chemokine (C–C motif) ligand 2 in acute pancreatitis.

Although U0126 and PD98059 have been widely applied in various disease conditions, to date there is no direct evidence that they—Let alone the new inhibitors mentioned above—Could treat DCM. Therefore, whether these two inhibitors and even other new selective ERK inhibitor can be used to prevent DCM remains to be investigated.

### 5.2. Exploiting Loss of Function Mutation of ERK1/2

Since ERK1/2 is associated with many pathological processes, the deletion of ERK1/2 might help us to alleviate individual disease conditions. Although there has been no study investigating the influence of ERK1/2 mutations in DCM models, there are a few studies exploring the influence of ERK1/2 deletion in the mice model of cardiac hypertrophy.

It is impossible to produce the ERK2 KO mouse by a traditional gene targeting method because of the embryonic lethality [82]. Ulm et al. generated mice with cardiomyocyte-specific deletion of ERK2 (ERK2^cko^) using the Cre-LoxP system. Protein expression or phosphorylation of cardiac ERK1 or MEK1/2 was not affected in this process. This cardiomyocyte-specific ERK2 null mouse model was then exposed to the short-term pathological hypertrophic stimulus. Transverse aortic constriction (TAC) and ISO in vivo or PE in vitro stimulus showed a mitigatory cardiac remodeling with decreasing cross-sectional area of individual myocytes, the downregulation of hypertrophic protein markers and less interstitial fibrosis by ERK2 deletion. However, cardiomyocyte apoptosis and decreased cardiac functional parameters such as fractional shortening (FS) were still present in these ERK2 null mice. These results demonstrated that ERK2 was responsible for cardiac hypertrophic growth. Knockout of ERK2 may play an essential role in treating pathological hypertrophic remodeling, despite inducing some apoptosis and cardiac dysfunction [83].

As for the salutary function of ERK1/2, cardio-protective influence of ERK1/2 through a pro-survival pathway in response to I/R injury was also evaluated by comparing ERK1 nullizygous gene-targeted mice (ERK1^−/−^), ERK2 heterozygous gene-targeted mice (ERK2^+/−^) and transgenic mice with activated MEK1 in their hearts. In this study, while transgenic MEK mice largely showed decreased I/R injury, loss of ERK1 did not significantly enhance MI in related measurement. However, when we consider that ERK2^+/−^ mice showed increased infarction size, DNA laddering, and TUNEL positivity compared with controls [84], ERK2 may be the target gene involved in the cardio-protective influence of ERK1/2 function during I/R injury.

A recent study challenges the hypothesis that ERK1/2 activation induces cardiac hypertrophy under different pathological stimulus. Purcell et al. found that mice lacking ERK1 and one ERK2 allele (ERK1^−/−^, ERK2^+/−^) showed a normal hypertrophic response to pressure overload and exercise stimuli. Since the residual activity of ERK2^+/−^ was sufficient to induce the required downstream pro-hypertrophic gene transcription, Purcell’s team tried to reduce the activity of ERK2 applying overexpressed phosphatase DUSP6, resulting in the near elimination of ERK1/2 activity in these mice at baseline, even after the stimulations of pressure overload, PE, ISO, or angiotensin II (Ang II). However, complete inhibition of ERK1/2 mediated by DUSP6 overexpression did not reduce cardiac hypertrophy after pressure overload stimulation, neuroendocrine agonist infusion, or physiologic exercise stimulation [48]. This study, which included overexpression of DUSP6, cannot support the hypothesis that cardiac hypertrophy could develop completely independent of ERK1/2, since DUSP6 may have other targets rather than ERK1/2. Furthermore, there is a notion that DUSP6 specifically inactivate ERK1/2 in the cytoplasm, while DUSP5 dephosphorylates nuclear ERK. Therefore, there is a possibility that nuclear ERK1/2 activity may still be present in DUSP6 overexpressed mice hearts.

To further understand the necessity of ERK1/2 signaling for cardiac hypertrophy, Purcell et al. generated cardiac-specific ERK1/2 targeted mice: ERK1^−/−^ ERK2^fl/fl-Cre^. The elimination of both ERK1 and ERK2 from the heart resulted in spontaneous eccentric hypertrophy with the dilatation of the heart, the elongation of the cardiomyocytes, decreased FS and early adult lethality resulting from heart failure. In addition, when they administrated AngII/PE infusion, ERK1^−/−^ ERK2^fl/fl-Cre^ mice showed a greater increase in left ventricular end-diastolic dimension and a greater decrease in FS compared with control mice. Next, they isolated adult cardiomyocytes from ERK1^−/−^ ERK2^fl/fl-Cre^ mice hearts and compared them with myocytes from activated MEK1 (aMEK1) transgenic mice. Remarkably, adult myocytes from ERK1^−/−^ ERK2^fl/fl-Cre^ mice were significantly longer, whereas aMEK1 myocytes were wider and shorter. These results suggested that ERK1/2 activation could result in concentric myocyte growth, whereas ERK1/2 deletion promotes an eccentric growth of myocyte. This study demonstrated for the first time that ERK1/2 signaling could play a key role in preventing eccentric cardiomyocyte growth and thus may produce protection from cardiac hypertrophy. Indeed, ERK1/2 double-null ventricular myocytes lengthened with aging and pathological stimulation to cause whole-organ eccentric growth. Interestingly, activation of ERK1/2 with MEK1 preferentially programmed concentric cardiac growth, while, at the same time, partially inhibiting eccentric growth. Taken together, these results suggest ERK1/2 signaling is necessary for facilitating a preferential type of cardiac growth in vivo, concentric hypertrophy that includes myocyte thickening and an addition of sarcomeres at the periphery while preventing eccentric growth and addition of sarcomeres in series [85].

In summary, from these results, we suggest that ERK2 plays a key role in the development of pathological hypertrophic remodeling as well as prevention of I/R injury. In contrast, both ERK1^−/−^ ERK2^+/−^ (3/4 alleles) and DUSP6-TG mice did not show a reduction in cardiac hypertrophy after different stimulations, which might result from incomplete deletion of ERK1/2 and/or nonspecific effects of DUSP6. Therefore, along with these two approaches to delete ERK1/2, other new selective and complete ERK1/2 double deletion animal models may be used to study the preventative effects against cardiac dysfunction and remain to be investigated.

## 6. ERK Phosphorylation Site

It is known that ERK1/2 are activated by MEK1/2-dependent phosphorylation in the TEY motif of the activation loop. However, Lorenz et al. found that there is another phosphorylation site of ERK2 at Thr188 (ERK1 at Thr208) that directs ERK1/2 to phosphorylate nuclear pro-hypertrophic target genes. They indicated that Thr188 autophosphorylation also required multiple steps, including activation of the whole Raf-MEK-ERK cascade, phosphorylation of the TEY motif, dimerization of ERK1/2, and binding to G protein βγ subunits released from activated G_q_. Like conventional phosphorylation in the TEY motif at threonine 202 and tyrosine 204, Thr188 autophosphorylation induced hypertrophy growth in isolated cardiomyocytes in mice upon stimulation of G_q_-coupled receptors or after aortic banding and in failing human hearts. From the study of transgenic mice examining Thr188 auto-phosphorylation, we now know that Thr188 phosphorylation plays a key role in ERK1/2-mediated cardiac hypertrophy. Blockage of Thr188 phosphorylation inhibited, whereas stimulation of Thr188 phosphorylation enhanced, TAC-induced hypertrophy and fibrosis [49].

Although ERK1/2 are thought to be the central mediators of cardiac hypertrophy and are regarded as potential therapeutic targets, however, the direct inhibition of ERK1/2 may also unexpectedly lead to exacerbated cardiomyocyte death and impaired heart function [86,87]. This complex phenomenon was still observed after blockage of Thr188 phosphorylation in ERK1/2. Ruppert et al. blocked the ERK2 Thr188 phosphorylation with the mutant ERK2 T188A. Whereas this type of mutation of ERK2 showed cardiomyocyte hypertrophy in the condition of PE and TAC in vitro and in vivo (it did not affect physiological cardiac growth and function), it blocked pathologic hypertrophy without increasing cardiomyocyte death [88]. Interestingly, Vidal et al. found that the absence of ERK Thr188 phosphorylation prevented nuclear accumulation of ERK1/2. Activation of nuclear ERK1/2, which is responsible for hypertrophic growth, was inhibited by the segregation of ERK1/2 in the cytosol [89]. In summary, these studies imply that a specific site of ERK1/2 activation at Thr188 may be responsible for the cardiac hypertrophy and fibrosis induced by different stimuli. Additionally, ERK Thr188 phosphorylation is required for the ERK1/2 nuclear localization that activates the pro-hypertrophic genes. In this case, ERK Thr188 phosphorylation might be a novel potential target for cardiac hypertrophy therapy.

## 7. HDAC Inhibitors Regulate ERK1/2 Activity in the Heart

HDACs are a large group of enzymes including four main classes: class I HDACs (HDAC 1, 2, 3, and 8), class II HDACs (HDAC 4, 5, 6, 7, 9, and 10), class III HDACs (sirtuins) and class IV HDAC (HDAC 11), all of which are responsible for the removal of the acetyl group from histones and thus influence expression of genes linked to the histone molecule [90]. Many studies suggest that HDAC inhibitors protect the heart against MI [91], I/R injury [92], and spontaneous hypertension [93]. Recently, HDAC inhibition has been reported to effectively attenuate DCM [94,95,96], which indicates that the HDAC might be a potential target of DCM. Interestingly, Majumdar et al. found that expression of phosphorylated ERK1/2, but not total ERK1/2, in H9c2 cells was significantly decreased by exposure to trichostatin A (TSA), an HDAC inhibitor, for 4–24 h [97]. Ferguson et al. reported that in neonatal rat ventricular myocytes treated with highly selective class I HDAC inhibitor MGCD0103, DUSP5 is highly induced to dephosphorylate and suppress nuclear ERK1/2 signaling, which results in potent inhibition of PE-induced hypertrophy [98]. Both HDAC inhibitors, such as TSA and valproic acid (VPA), and depletion of HDAC3 by RNA interference significantly block the activation of ERK1/2 and its downstream target 90 kDa Ribosomal S6 kinases (p90RSK) and upstream kinase c-Raf by TGF-β [99]. These findings suggest that HDACs, and in particular HDAC3 are required for activation of the ERK1/2 signaling pathways. Furthermore, Williams et al. reported that Ang II-induced cardiac interstitial collagen deposition is completely abolished by pan-HDAC inhibition with scriptaid or class I HDAC-selective inhibition with MGCD0103, the effect of which might be mediated, at least in part, by suppressing ERK1/2 signaling [100]. This result might imply that class I HDACs and their substrate ERK1/2 signaling cascade might be the target to modulate pathological cardiac fibrosis and hypertrophy that may lead to the development of DCM.

In summary, these findings suggest that inhibition of ERK1/2 mediated by HDAC inhibitor might be one of the important regulatory mechanisms of hypertrophy induced by different stimuli, which might provide a new target for therapies against cardiac complications induced by diabetes.

## 8. MicroRNAs Regulates ERK1/2 Activity in Diabetic Heart

miRNAs are the small noncoding RNAs that bind to sequence-specific mRNA and consequently inhibit translation. Emerging evidence reveals the crucial role of miRNAs in the development or suppression of cardiovascular diseases. It is reported that some miRNAs are involved in the regulation of ERK1/2 in diabetic conditions. Here, as shown in Table 2, we will summarize the influence of these miRNAs in regulating ERK1/2 activity in some pathological models and during intervention procedures of DCM.

The marked increase in the level of expression of miR-200c was found in aortas and kidneys of diabetic *db*/*db* mice compared with nondiabetic mice [101,105]. Overexpression of miR-200c led to impairment of endothelium-dependent relaxations (EDRs) in nondiabetic mouse aortas, while suppression of miR-200c by anti-miR-200c enhanced EDRs in diabetic *db*/*db* mice [105]. Therefore, it might be possible that similar putative negative influence regulating ERK1/2 is expected to be found in miR-200c expression in diabetic hearts. In human umbilical vein endothelial cells (HUVECs) treated with high glucose, overexpression of miR-320 can reduce expression of ET-1, vascular endothelial growth factor (VEGF), and fibronectin through inhibiting ERK1/2 to alleviate cardiac injury induced by diabetes [102]. It has been reported that miR-133a was down-regulated in pressure overload hypertrophy [103] and diabetes [106], while miR-133a overexpression, reducing ERK1/2 activation, can alleviate cardiac fibrosis. The fact that this reduction in activation of ERK1/2 by overexpression of miR-133a may improve DCM implies that ERK1/2 can be regulated by the miR-133a in diabetic hearts [106]. In addition, both miR-1 and miR-6 are involved in the development of DCM, which has been demonstrated in STZ-induced diabetic hearts, high glucose-treated neonatal rat ventricular cardiomyocytes (NRVCs), and H9c2 cells [104,107]. The overexpression of miR-1 and miR-6 in HG-treated H9c2 cells is mediated by MEK1/2, which can be abolished by the MEK1/2 inhibitor PD98059 [104]. In summary, a variety of miRNAs has been found to regulate ERK1/2-mediated cardiac apoptosis and remodeling in diabetic hearts. The role of miRNAs in DCM and ERK1/2 induced heart disease in diabetes is a vibrant topic and needs further exploration.

## 9. Conclusions

One of three well-defined subgroups of MAPKs, ERK MAPK, is involved in both cell growth and cell death, the tight regulation of which, therefore, is crucial in determining cell fate. ROS, important injury, and stimulus leading to both physiologic adaptation and pathologic disease conditions have been reported to activate ERKs, but the mechanism by which ROS can be activated by ERKs are unclear. Actually, activation of the ERK1/2 signaling cascade has long been implicated in mediating most stress stimuli-induced cardiac hypertrophy. Therefore, many compounds are developed that inhibit the ERK1/2 pathway such as MEK-ERK inhibitors, PD98059 and U0126 and that thus potentially attenuate cardiac growth and proliferation. Recently, some selective ERK1/2 inhibitors were developed for fear of the potential side-effects of the MEK-ERK inhibitor. ERK1/2 double KO mice blocking of the phosphorylation site of ERK1/2 at threonine 202 and tyrosine 204 have been introduced to define the differential role of ERK1/2 in exerting in the heart. In addition, a new type of ERK1/2 inhibitor blocking phosphorylation site at Thr188 has also been discovered to inhibit ERK1/2 to phosphorylate nuclear targets such as those known to cause cardiac hypertrophy specifically. Despite the pro-apoptotic role of ERK1/2 in most diabetes settings, the pro-survival (anti-apoptotic) effect of ERK1/2 was noted which protects against the cardiac injury induced by I/R and MI in the diabetic heart. Moreover, FGF21-mediated protection through ERK1/2 activation against DCM development, myocardial I/R injury, and ISO-induced cardiac hypertrophy has been demonstrated. Although the precise mechanisms of the ERK1/2 signaling cascade in the regulation of cell survival and death are not yet fully understood, it is widely accepted that the duration, extent, and subcellular compartment of ERK1/2 activation are vital to differential biological effects of ERK1/2. Finally, research on epigenetic regulation by histone deacetylases (HDACs) and/or the HDACs inhibitor and miRNA, along with the strategies associated with the ERK1/2 pathway modulation, has attracted intense interest in preventing DCM.

## Figures and Tables

**Table 1:** Inhibitors of MEK/ERK.

Inhibitors	Isoforms	Model	Response	References
U0126	MEK/ERK	Cardiomyocyte treated with HG and animal model of myocardial I/R injury	Alleviated the HG and I/R-induced cardiomyocyte injury	[58,81]
PD98059	MEK/ERK	H9c2 cell treated with AGEs and diabetic mice induced by STZ	Prevented cardiomyocyte hypertrophy and cardiac remodeling and apoptosis of diabetic mice	[23,63]
FR 180204	ERK1/2	WT and Pak-1-KO mice treated with ISO	Inhibited the cardiac hypertrophy	[75]
SCH772984	ERK1/2	pancreatic cancer clinical samples	Anti-cancer	[78]
VX-11e	ERK1/2	Patient derived xenograft models	Anti-cancer	[79]
GDC-0994	ERK1/2	Rats treated with SFAs or UFAs	Reduced inflammatory response	[80]

HG, high glucose; AGEs, advanced glycation end products; STZ, streptozocin; Pak-1-KO, p21-activated kinase-1 knockout; ISO, isoproterenol; SFAs, saturated fatty acids; UFAs, unsaturated fatty acids.

**Table 2:** MicroRNAs involved in an ERK1/2 activity.

MicroRNA	Location	Model	Response	References
miR-200c	Upstream	*db*/*db* T2DM	Increase aorta endothelial dysfunction	[101]
miR-320	Upstream	High glucose treated HUVECs	Reduced expression of ET-1, VEGF, and FN	[102]
miR-133a	Upstream	STZ-induced diabetes	Prevent cardiac fibrosis	[103]
miR-1 and 6	Up stream	STZ-induced diabetes and high glucose treated NRVCs	Increase cardiomyocyte apoptosis	[104]

Location refers to being upstream or downstream of ERK1/2; STZ, streptozotocin; T2DM, type-2 diabetes mellitus; HUVEC, human umbilical vein endothelial cells; NRVCs, neonatal rat ventricular cardiomyocytes; ET-1, endothelin 1; VEGF, vascular endothelial growth factor; FN, fibronectin.

## Abbreviations

AGEs	advanced glycation end products
Ang II	angiotensin II
DCM	diabetic cardiomyopathy
DUSPs	dual-specificity phosphatases
ERK1/2	extracellular signal-regulated kinase 1/2
ET-1	endothelin 1
FGF21	fibroblast growth factor 21
FN	fibronectin
FS	fractional shortening
GPCR	G-protein coupled receptor
HDAC	histone deacetylase
HG	high glucose
HUVECs	human umbilical vein endothelial cells
I/R	ischemia/reperfusion
ISO	isoproterenol
JNK	c-Jun N-terminal protein kinase
MAPK	mitogen-activated protein kinase
MEK	MAPK kinase
MI	myocardial infarction
miRNA	microRNA
MKPs	MAPK phosphatases
MMP	mitochondrial membrane potential
NO	nitric oxide
NRVCs	neonatal rat ventricular cardiomyocytes
Pak-1	p21-activated kinase-1
PE	phenylephrine
PP2A	protein phosphatase 2A
ROS	reactive oxygen species
SFAs	saturated fatty acids
SOD	superoxide dismutase
STZ	streptozocin
T1DM	type 1 diabetes mellitus
T2DM	type 2 diabetes mellitus
TAC	transverse aortic constriction
TGF-β	transforming growth factor beta
TSA	trichostatin A
UFAs	unsaturated fatty acids
VEGF	vascular endothelial growth factor
